# Variations in the structure and reactivity of thioester functionalized self-assembled monolayers and their use for controlled surface modification

**DOI:** 10.3762/bjnano.3.24

**Published:** 2012-03-09

**Authors:** Inbal Aped, Yacov Mazuz, Chaim N Sukenik

**Affiliations:** 1 Department of Chemistry and Institute for Nanotechnology and Advanced Materials, Bar-Ilan University, Ramat-Gan, Israel 52900

**Keywords:** siloxane-anchored self-assembled monolayers, sulfonated interfaces, surface chemistry

## Abstract

Thioester-functionalized, siloxane-anchored, self-assembled monolayers provide a powerful tool for controlling the chemical and physical properties of surfaces. The thioester moiety is relatively stable to long-term storage and its structure can be systematically varied so as to provide a well-defined range of reactivity and wetting properties. The oxidation of thioesters with different-chain-length acyl groups allows for very hydrophobic surfaces to be transformed into very hydrophilic, sulfonic acid-bearing, surfaces. Systematic variation in the length of the polymethylene chain has also allowed us to examine how imbedding reaction sites at various depths in a densely packed monolayer changes their reactivity. π-Systems (benzene and thiophene) conjugated to the thioester carbonyl enable the facile creation of photoreactive surfaces that are able to use light of different wavelengths. These elements of structural diversity combine with the utility of the hydrophilic, strongly negatively charged sulfonate-bearing surface to constitute an important approach to systematic surface modification.

## Introduction

Functionalized self-assembled monolayers (SAMs) provide powerful tools for conveniently adjusting the composition and chemistry of solid interfaces. First introduced by Jacob Sagiv and co-workers [1–3], siloxane-anchored SAMs have been used to modify the wetting and composition of variously hydroxylated surfaces. In situ chemical transformations of the SAM surfaces provide an additional dimension to the versatility and utility of the SAMs [4–7].

Our laboratory has reported in situ transformations of siloxane-anchored SAMs in which SAM surface functionality was changed from benzene rings to arylsulfonic acids [8–9], from nitrate esters to hydroxyls [10], and from carboxylate esters to carboxylic acids [11–12]. All three of these functionalized surfaces could not have been deposited directly since the requisite silanes would not have been stable. Layer-by-layer [13] and modular assembly [14] of sulfonic acid surfaces with a lower degree of order and uniformity has also been reported.

A striking example of in situ SAM transformations is based on the initial deposition of thioacetate-bearing monolayers and their in situ conversion to sulfonic acid surfaces [15]. This transformation provides the basis for surface patterning of the monolayer and for its use as a patterned template for inorganic oxide deposition [16]. The work reported herein extends this chemistry in two important directions. In one instance, thioesters with acyl components of varying chain length are shown to provide a tool for varying the initial hydrophobicity of the monolayer surface from medium hydrophobicity (water contact angles of about 70°) to very hydrophobic (water contact angles >110°). Each of these thioesters can be converted into sulfonic acids so as to provide fully wetted surfaces. The systematic variation in molecular chain length that produced the steadily changing hydrophobicity also allowed an examination of how the imbedding of reaction sites at various depths within a well-packed monolayer affects their reactivity. In another variation of monolayer structure, a set of thioesters with different aromatic rings conjugated to the carbonyl facilitate efficient photocleavage using longer wavelength light such that the photo-oxidation of the thioesters to sulfonic acid can be achieved with light of wavelength >300 nm.

**Figure 1:**
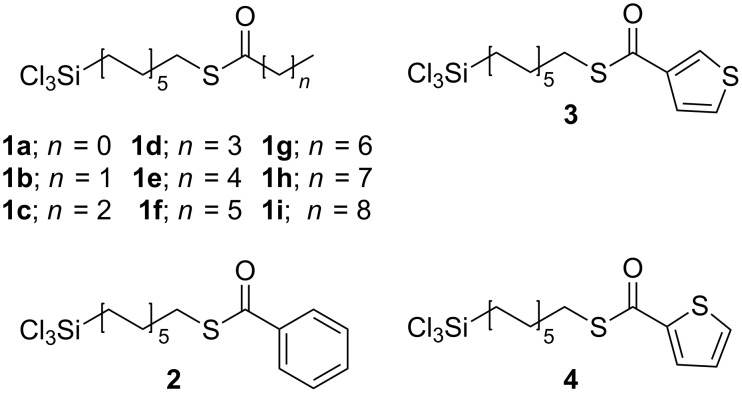
Trichlorosilyl thioesters.

We have synthesized a series of thioesters (Figure 1) that were designed to provide a range of hydrophobicities (**1a–i**) and a range of photoreactivities (**2–4**). These trichlorosilanes have been used to make siloxane-anchored monolayers on silicon wafers and quartz. The siloxane-anchored SAMs based on these materials, their tunable wetting properties and their in situ chemical transformations are the focus of this report.

## Experimental

### General methods and materials

#### Materials

Reagents and solvents were obtained from Sigma-Aldrich, Acros Organics, Fluka, Bio-Lab Ltd. or Merck. They were all used as received unless otherwise indicated. Water was deionized and then distilled in an all-glass apparatus. Column chromatography used silica gel 60 (230**–**400 mesh). Silicon wafers were obtained from Virginia Semiconductor (n-type; undoped, <100>, >1000 Ω·cm). Quartz substrates were obtained from Quarzschmelze Ilmenau.

#### Analytical Methods

Unless otherwise indicated, NMR spectra were obtained on a Bruker DPX 300 spectrometer (^1^H NMR at 300 MHz; ^13^C NMR at 75 MHz). Some were performed on a Bruker DPX 200 spectrometer (^1^H NMR at 200 MHz; ^13^C NMR at 50 MHz). The spectra are reported in ppm units (δ) and are referenced to TMS at 0 ppm for ^1^H NMR and to CDCl_3_ at 77.160 ppm for ^13^C NMR. UV spectra (200–800 nm) were measured on a Cary Model 100 spectrometer (in double-beam transmission mode). Spectra of the as-deposited films were collected by using quartz slides. Spectra were run against a reference sample of the same quartz without the deposited films. Mass spectra were recorded on a Finnigan Model 400 mass spectrometer, by using chemical ionization (CI) with methane as the reagent gas unless otherwise indicated. Contact angle goniometry, spectroscopic ellipsometry, XPS, ATR–FTIR, were all carried out as previously described [11–12].

#### Syntheses

ω-Undecenylbromide was prepared as follows: In a round-bottom flask (500 mL) equipped with a magnetic stirring bar were placed CH_2_Cl_2_ (100 mL), commercial undecen-1-ol (12 g, 70.5 mmol) and triphenylphosphine (20.2 g, 77.0 mmol). The flask was cooled to 0 °C. While being stirred vigorously, tetrabromomethane (23.37 g, 70.5 mmol) was added slowly. After the addition, the mixture was stirred for 2 h and the CH_2_Cl_2_ was removed on a rotovap. The residual white paste was broken up and stirred with hexane (100 mL) and filtered into a round-bottom flask (250 mL). The hexane was removed on a rotovap. The crude product was purified by flash chromatography (hexane): Yield 15.53 g (94.5%). NMR analyses match those reported previously in the literature [6].

The preparation of (*S*)-undec-10-enyl thioacetate from ω-undecenylbromide followed the previously published procedure [17]. ω-Undecenyl thiol was prepared by acid hydrolysis of the thioacetate, as follows: In a round-bottom flask (250 mL) equipped with a magnetic stirring bar and a reflux condenser were placed methanol (135 mL) and HCl (15 mL, 37%). To this was added (*S*)-undec-10-enyl thioacetate (9 g, 39.4 mmol) and the mixture was heated under reflux overnight. The heating was stopped and the solvent was removed on a rotovap. Hexane (100 mL) was added, and the solution was extracted with water (50 mL) and brine (50 mL). The hexane was dried over MgSO_4_ and filtered, and the solvent was removed on a rotovap. The crude ω-undecenyl thiol was purified by flash chromatography (hexane): Yield 6.02 g (82%); ^1^H NMR δ 1.20–1.47 (m, 13H), 1.61 (m, 2H), 2.04 (m, 2H), 2.52 (q, *J* = 7.5 Hz, 2H), 4.93 (m, 2H), 5.81 (ddt, *J* = 6.6, 10.2, 17 Hz, 1H); ^13^C NMR δ 24.80, 28.51, 29.06, 29.20, 29.24, 29.56, 29.59, 33.95, 34.19, 114.27, 139.36.

The general procedure for the conversion of ω-undecenyl thiol into the thioester–olefin precursors for compounds **1b**–**i**, **2**, **3** and **4** is as follows: In a dry, round-bottom flask equipped with a magnetic stirring bar were placed ω-undecenyl thiol (*x* mmol) and NEt_3_ (6*x* mmol) in dry THF (54*x* mmol). The flask was cooled to 0 °C, and the appropriate acid chloride (1.01*x* mmol) was added slowly. After 2 h the reaction mixture was warmed to room temperature and the solvent was removed on a rotovap. Hexane (100 mL) was added and the solution was extracted with water (50 mL), 20% NaHCO_3_ (50 mL) and brine (50 mL). The hexane was dried over MgSO_4_, filtered and the solvent was removed on a rotovap. The aliphatic thioesters were purified by flash chromatography (5% EtOAc, 95% hexane), while vacuum distillation was used to purify the benzoyl and thiophenyl thioesters. Isolated yields, ^1^H and ^13^C NMR, and exact mass MS data for each of the olefin-thioesters are summarized in Supporting Information File 1.

The general procedure for the conversion of the various olefin thioesters into trichlorosilanes **1**, **2**, **3**, and **4** is as follows: The olefin thioester (1–2 mL), HSiCl_3_ (6 mL), and a solution of H_2_PtCl_6_·6H_2_O in iPrOH (10–20 µL, 4%; dried over 4 Å molecular sieves and distilled) were placed in a pressure tube (20 mL) containing a magnetic stirring bar. All reagents were handled in a nitrogen atmosphere. The tube was sealed and transferred to an oil bath maintained at 60–80 °C, in which it was heated for 16–40 h (the specific temperatures and times are given in Supporting Information File 1). The progress of the reaction was monitored by the disappearance of the olefinic protons in the ^1^H NMR. After the reaction was complete, the contents of the tube were transferred to a round-bottom flask (25 mL) under a nitrogen atmosphere. Excess HSiCl_3_ was distilled off and the product was isolated by Kugelrohr distillation. The isolated yields and NMR data for each of the trichlorosilanes is summarized in Supporting Information File 1.

#### Monolayer preparation

Silicon wafers (for ellipsometry and ATR–FTIR measurements) and quartz wafers (for UV and XPS measurements) were cleaned and activated as previously reported [12] and used as substrates for depositing siloxane-anchored SAMs based on compounds **1**–**4**. The SAMs were characterized by contact angle, ATR–FTIR, UV–vis, ellipsometry, and XPS. These characterization tools were applied (as previously reported [12]) both on the directly deposited SAMs and on those that had been subjected to the oxidation reactions reported herein.

### General procedures for in situ oxidation of thioester SAMs

#### Oxidation using aqueous OXONE

A saturated solution of OXONE (potassium peroxomonosulfate, extra pure, min. 4.5% active oxygen; Acros Organics) in water was prepared. The thioester SAM-bearing substrates were immersed in the OXONE solution for times of up to 10 h (see Table 2 below), at room temperature [15]. The substrates were withdrawn from the solution, rinsed with doubly distilled water, and dried under a stream of filtered nitrogen.

#### UV-C irradiation in air

A UV lamp (narrow-band irradiation centered on 254 nm, 6 W lamp) was held 2 cm from the surface of the substrate for 1 h for each side (in ambient air). The oxidized surface was rinsed with doubly distilled water and dried with a stream of filtered nitrogen. In some instances, the photoreacted surfaces were rinsed with CHCl_3_ and EtOH before the final water rinse. The consequences of these rinses with organic solvents will be discussed below.

#### UV-A irradiation in air

Quartz test-tubes were used as holders for silicon and quartz wafers coated with SAMs based on **1a**, **2**, **3** and **4**. The test-tubes were placed in the middle of a Luzchem model LZC4 photoreactor (8 UV-A lamps, HITACHI FL8BL-B, emission 320–400 nm, peak emission at 360 nm) such that the lamps completely surrounded the samples. Irradiation times were up to 132 h, at 24–28 °C. After irradiation, the substrates were withdrawn from the reactor, rinsed with doubly distilled water, and dried under a stream of filtered nitrogen.

## Results

### SAM preparation

Trichlorosilane **1a** was prepared by a method similar to that reported for its longer chain analogue [15], and compounds **1b**–**i, 2, 3** and **4** were all produced by hydrosilylation of a terminal olefin that was obtained by acylation of ω-undecenyl thiol, which had been prepared in three steps from commercial ω-undecenol. All of the trichlorosilanes were purified by distillation and deposited as siloxane-anchored SAMs.

### SAM characterization

ATR–FTIR characterization of these SAMs focused on the vibrational frequencies of the carbonyl groups and of the methylene units in each of the polymethylene chains (Table 1). The carbonyl stretches of the alkyl thioesters are all in the range of 1690–1696 cm^−1^. The conjugation in **2**, **3**, and **4** reduces the stretching frequency to 1654–1662 cm^−1^. In all cases, the disappearance of the carbonyl stretching frequency is a straightforward diagnostic for the oxidative cleavage. The methylene stretching frequencies for all of the thioester SAMs are typical of monolayers with low crystallinity in their chain packing [18–19].

**Table 1:** FTIR data for SAMs based on compound **1**–**4**.

SAM	ATR–FTIR (cm^−1^)		
	CH_2_ antisymmetric	CH_2_ symmetric	C=O

**1a**	2922	2851	1695
**1b**	2922	2852	1696
**1c**	2923	2852	1693
**1d**	2922	2851	1691
**1e**	2922	2851	1691
**1f**	2923	2852	1691
**1g**	2922	2851	1690
**1h**	2922	2852	1691
**1i**	2921	2851	1690
**2**	2922	2851	1662
**3**	2922	2851	1660
**4**	2922	2851	1654

**Figure 2:**
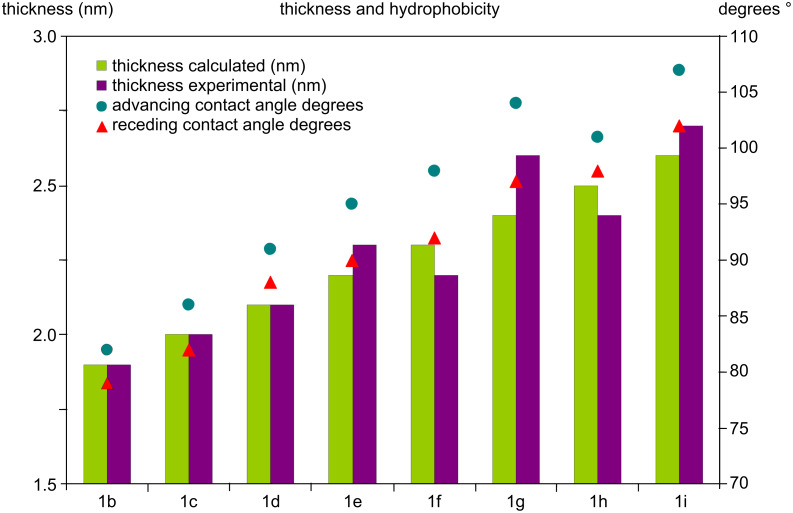
Thickness and contact angles (advancing/receding) for SAMs based on compounds **1b**–**i**.

Compounds **1** represent a homologous series whose variable chain length systematically changes the film thickness and surface hydrophobicity. The thicknesses (±0.2 nm) and wetting behaviors (±3°) of the members of the series with 1–8 methylene units in the acyl chain are summarized in Figure 2 so as to highlight the steady increase in monolayer thickness (calculated based on fully extended alkyl chain and observed by ellipsometry) and hydrophobicity. The SAM based on **1a** (no methylene units) is relatively hydrophilic (contact angle 75°/67°) even when compared to the analogue containing only one methylene unit, **1b** (82°/79°). This reflects both the shorter alkyl chain and the closer proximity of its carbonyl groups to the SAM surface. The contact angles for SAMs based on compounds **2** (78°/72°), **3** (83°/75°) and **4** (80°/72°) are reasonable for such terminal aryl groups.

**Figure 3:**
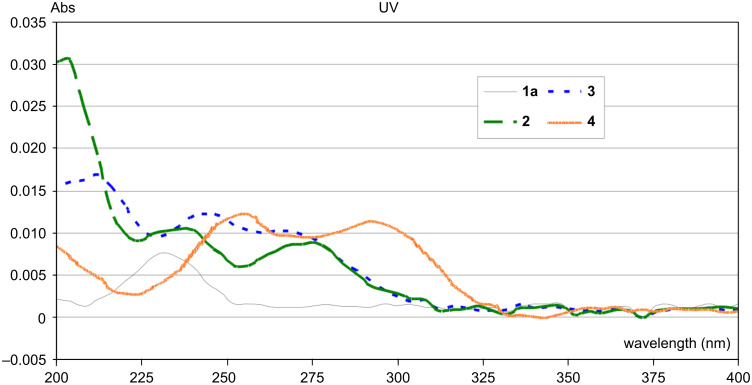
UV–vis spectra of SAMs of compounds **1a**, **2**, **3** and **4**.

The UV–vis spectra of compounds **1a, 2, 3** and **4** are compared in Figure 3. The spectra of compounds **1b**–**i** are all comparable to that of **1a**. These spectral features provide the basis for their varying interactions with the different wavelengths of light used for SAM photo-oxidation.

### In situ SAM oxidations

Monolayers of compounds **1**–**4** were all subjected to treatment with aqueous OXONE solutions under ambient conditions. In all cases, the starting monolayer is comprised of siloxane-anchored units with 11 methylene groups that terminate in a thioester (Si–(CH_2_)_11_–SCOR), and the result is always the same sulfonate-decorated SAM, tethered through a chain of 11 methylenes (Si–(CH_2_)_11_–SO_3_H). After reaction times of 2–10 h (Table 2), all of the surfaces became very hydrophilic, with water contact angles of <25°.

**Table 2:** Reaction times and methylene loss (based on ATR–FTIR integration) for OXONE oxidation of SAMs of compounds **1**–**4**; all surfaces became highly hydrophilic (water contact angles <25°).

SAM	reaction time (h)	percent of remaining methylene FTIR peak intensity	
		calculated	observed

**1a**	2.0	100%	92%
**1b**	2.0	92%	87%
**1c**	2.0	85%	86%
**1d**	2.5	79%	73%
**1e**	4.0	73%	78%
**1f**	5.0	69%	77%
**1g**	6.0	65%	62%
**1h**	7.0	61%	66%
**1i**	10.0	58%	51%
**2**	6.0	100%	98%
**3**	6.0	100%	94%
**4**	6.0	100%	108%

In SAMs based on compounds **1b**–**i** the intensity of the methylene peaks in the IR decreases after oxidation as a result of the removal of the acyl chain. We can compare the observed methylene peak intensity to that which is expected based on the number of methylenes that remain relative to the original total number of methylenes. The expected value of this ratio if only the 11-carbon polymethylene tether remained and all of the methylene units of the acyl chain were removed, as well as the observed integrated ratio of the antisymmetric methylene peaks before and after oxidation, are shown in Table 2. Since the oxidation of SAMs based on **1a**, **2**, **3** and **4** removes no methylene units, it is expected that there should be little or no change in the methylene peak intensity. The observed peak intensity matches the expected value (±10%).

**Figure 4:**
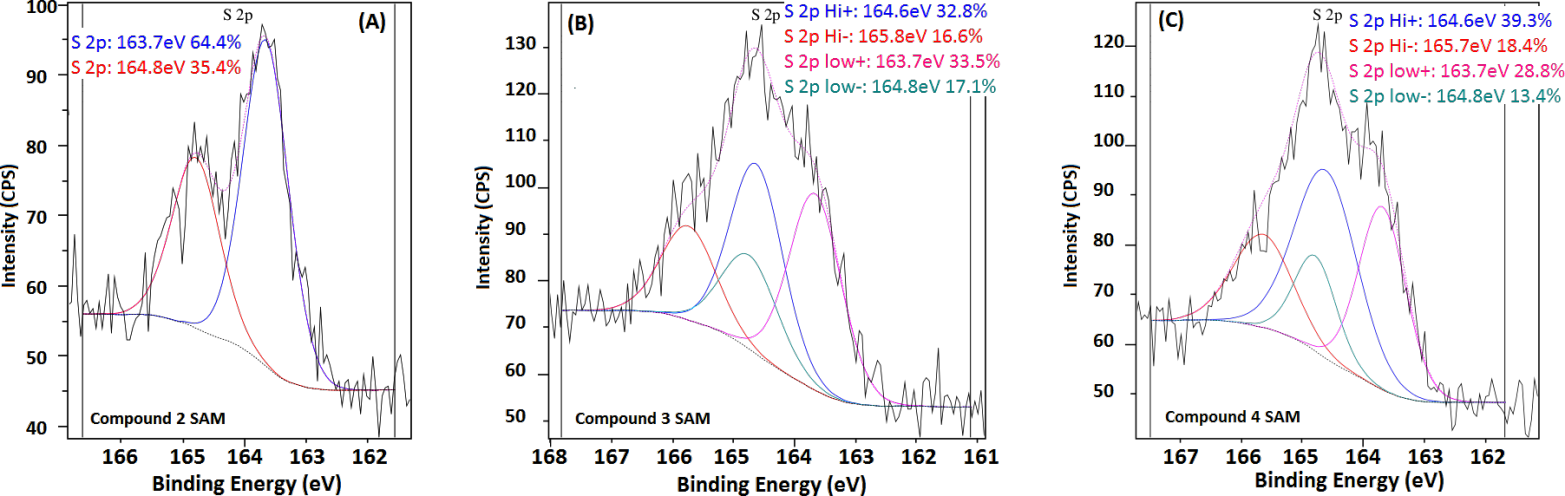
Representative sulfur XPS analyses of the SAMs of compounds **2** (**A**), **3** (**B**) and **4** (**C**).

The oxidation of the thioester-bearing SAMs was also followed by XPS. SAMs of compounds **1** and **2** showed peaks corresponding to the expected divalent sulfur of the thioester at 163.8 ± 0.2 eV and 164.8 ± 0.2 eV in the expected 2:1 ratio (±10%), see for example Figure 4A. The additional (thiophene) sulfur in both compounds **3** and **4** leads to a broad, merged signal (Figure 4B and Figure 4C). Deconvolution reveals the thiophene sulfurs at 164.5 ± 0.2 eV and 165.6 ± 0.2 eV. The overlap among the four peaks in the spectra, together with their inherently problematic signal-to-noise ratio, leads to a situation in which the expected 2:1 peak intensity ratio for each sulfur and the expected 1:1 ratio for the two kinds of sulfurs in a single thiophene-bearing SAM show error bars of as much as 30%. Nevertheless, the XPS result confirms the presence of the thioester and thiophene sulfurs and attests to their complete disappearance (in all cases) upon oxidation to a sulfonic acid SAM (in which the one sulfur is at 168.2 ± 0.2 eV and 169.9 ± 0.2 eV).

Finally, the photo-oxidations of the various types of thioesters were compared by using UV radiation of different wavelengths. A lamp whose output was around 254 nm was used for irradiation at shorter wavelengths. This was compared to irradiations done with a broad-spectrum, longer-wavelength UV lamp (UV-A, 320–400 nm). Since the UV–vis absorption spectra of all compounds **1** were the same, only **1a** was used in the comparisons to the photo-oxidation behavior of compounds **2**–**4** at longer wavelength.

The SAMs with varying alkyl chain lengths (based on compounds **1b**–**i**) were subjected to photo-oxidation at 254 nm. Table 3 shows the changes in their water contact angle upon photochemical reaction in air. The removal of the acyl methylenes (as per the above discussion of the OXONE oxidation results in Table 2) is also shown. The completeness of the photocleavage is attested to by the fact that after an oxidation time of 2 h (1 h on each side) the carbonyl and methyl peaks in the IR disappeared and the intensities of the methylene signals were reduced by the amount expected for each chain length. However, the surfaces achieved were not as hydrophilic as expected. The unexpectedly high contact angles after oxidation, and their possible relationship to solvent induced surface reorganization and/or residual long-chain contaminants, will be addressed in the Discussion section.

**Table 3:** Contact angles and methylene loss (based on ATR–FTIR integration ratio, calculated and observed) before and after irradiation of SAMs based on compounds **1b**–**i**.

SAM	contact-angle measurement adv[°]/rec[°]		percent of remaining methylene FTIR peak intensity	
	before irradiation	after irradiation	calculated	observed

**1b**	82/79	35/<20	92%	82%
**1c**	86/82	60/40	85%	81%
**1d**	91/88	48/<20	79%	72%
**1e**	95/90	44/37	73%	72%
**1f**	98/92	70/49	69%	75%
**1g**	104/97	62/39	65%	69%
**1h**	101/98	65/43	61%	67%
**1i**	107/102	76/65	58%	67%

The irradiation with 254 nm light was also applied to monolayers based on compounds **1a, 2, 3** and **4**. Following the experience with SAMs based on compounds **1b**–**i**, and the fact that both benzoic acid and its thiophene analogues are more water soluble than the long-chain aliphatic acids, the rinsing procedure was changed so as to only use water. In this way, the complete photocleavage suggested by the disappearance of the carbonyl in the IR was accompanied by the formation of a fully wetted surface (contact angles <25°) for all of the SAMs based on compounds **1a, 2, 3** and **4**.

**Figure 5:**
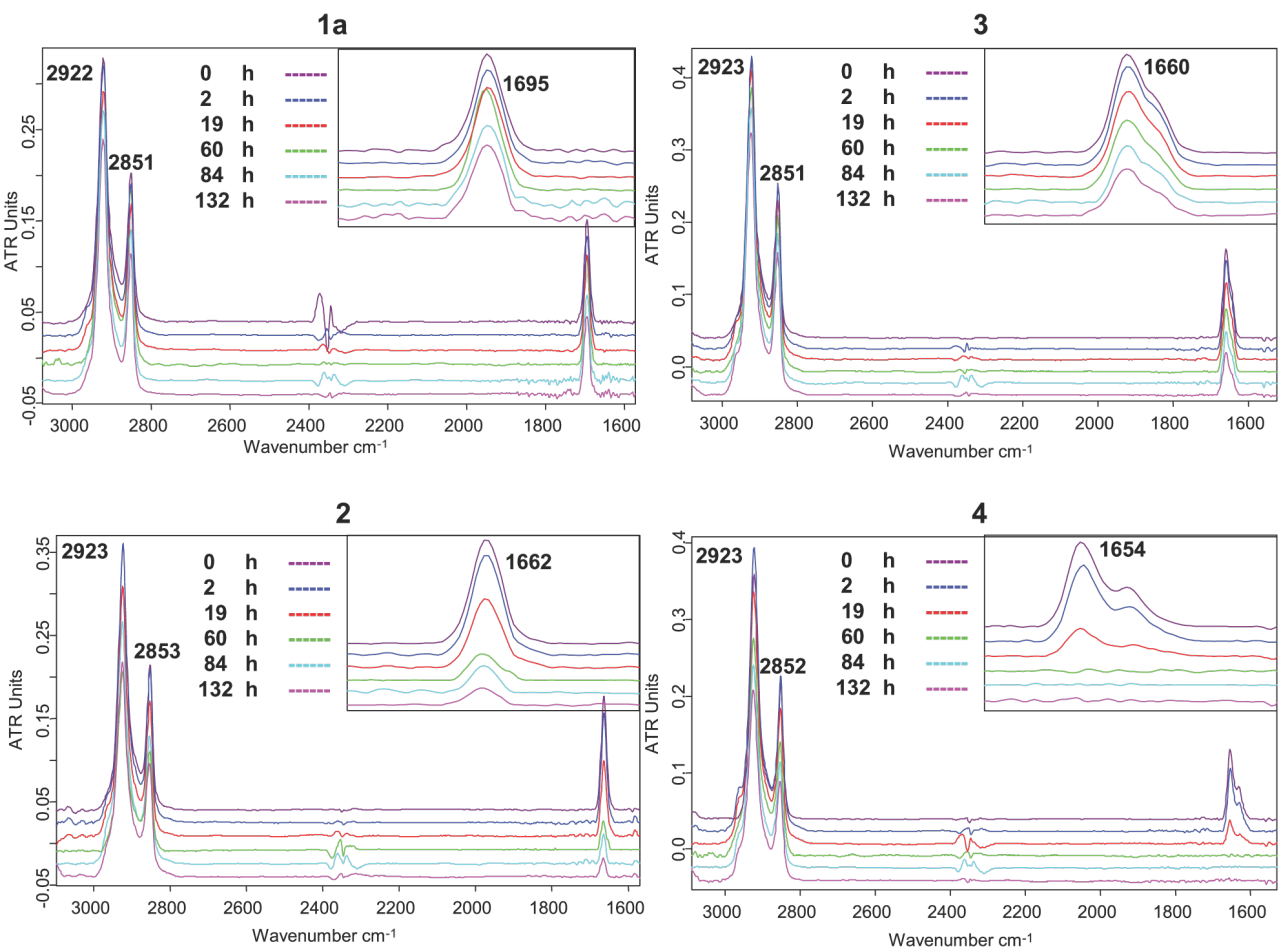
ATR–FTIR spectra of SAMs of compounds **1a**, **2**, **3** and **4**, as deposited, and after oxidation with UV-A in air for 2 h, 19 h, 60 h, 84 h and 132 h.

Photo-oxidations of SAMs based on compounds **1a, 2, 3** and **4** were also carried out by using a UV-A (320–400 nm) light source and exposure times of up to 132 h. These experiments are summarized in Figure 5. It is clear that the acetyl group in **1a** is not cleaved by the longer wavelength light, even after 132 h. SAMs based on compounds **2** and **3** show some photocleavage under these conditions, but the process is slow and never goes to completion. Their response to the longer wavelength light is anticipated by the fact that the UV-A light only has significant intensity at wavelengths longer than 320 nm, at which **2** and **3** do not absorb. On the other hand, SAMs based on compound **4** show an intense absorption peak at 290 nm and an absorption tail that extends to slightly beyond 325 nm. They undergo effective photocleavage even at longer wavelength.

## Discussion

In previous work with a longer chain analogue of **1a** (in which the thioacetyl group is connected to a 16-carbon chain instead of the 11 carbons in **1a**) [15,20], we reported the photoconversion of a thioacetate-decorated SAM to a sulfonated surface by UV irradiation in air. In that case, the initially deposited thioacetate-functionalized SAM had more closely packed alkyl chains (FTIR: methylene stretching frequencies 2919 and 2850 cm^−1^ versus the 2922–2923 cm^−1^ and 2851–2852 cm^−1^ of **1a**–**h**). Only **1i** displays some level of crystalline order (with methylene values of 2921/2851 cm^−1^), and even that is not as ordered as the C_16_ system [15,20].

The important conclusion from the oxidation of the acetylated thiols with the two different chain lengths is that both systems provide a sulfonated surface that is fully wetted. The acetyl-derived byproduct is easily removed by rinsing with water, and the resulting surface is hydrophilic. We note that while the oxidation of the longer chain thioesters (**1b**–**i**) with OXONE takes longer (as indicated by reaction times in Table 2), as would be expected for the more hydrophobic starting SAMs, the longer chain byproducts are successfully washed away and the resulting surface is also fully wetted. The slowing of the reaction with OXONE with increasing numbers of methylene units is reminiscent of what was seen by Sagiv et al. [21–23] for permanganate oxidation in which an olefin at the monolayer surface was oxidized much faster than an olefin within the monolayer. The fact that the monolayers reported herein are somewhat less well-packed than those reported in the permanganate oxidation study may be responsible for the fact that the differences in reactivity observed herein are smaller than those reported for the permanganate oxidation.

A problem with the longer chain acyl units is seen in their photo-oxidation. In that case, there is no evidence for a slowing of the reaction based on the rate of disappearance of the carbonyl, but the high degree of hydrophilicity that is achieved with aqueous OXONE is not obtained in these longer chain systems. It seems that there is a buildup of longer chain byproducts that need organic solvents to effectively remove them. However, the exposure of the high-free-energy sulfonated surface to organic solvent leads to surface reorganization and loss of hydrophilicity. Thus, in order to take advantage of the enormous change in surface wetting achieved by the oxidation of a system such as **1g**–**i** (from a water contact angle of over 100° to a fully wetted surface), oxidation in aqueous OXONE is most effective.

Beyond the impact of changing the chain length on the chemistry described above, we have also established a clear wavelength dependence on the photo-oxidation of the thioesters. The reactions of the benzene and thiophene derivatives are notable for a number of reasons. Firstly, the aromatic ring does not interfere with the chemistry described above. The reactivity of the thioester is not undermined (despite a small retardation of the OXONE reaction), either by the bulk of the aromatic rings or by the reduced electrophilicity, which is typical of conjugated carbonyl groups.

We also note the wavelength dependence of the photochemistry reported herein. The longer wavelength absorption of the conjugated chromophore is clearly a first step towards a system that could be photoreacted with longer wavelength light. This would provide a route to photopatterned sulfonate surfaces, in which the irradiation could be performed through regular glass or Pyrex, i.e., media that are not transparent to shorter wavelength UV radiation.

## Conclusion

Monolayers based on various thioacetate derivatives have been shown to provide useful control over surface wetting. The initially deposited monolayers are stable surfaces whose hydrophobicity can be systematically varied based on the length of the alkyl chain of the acyl moiety. Variously conjugated versions of the acyl moiety provide useful wavelength control over the photochemistry of the thioesters. The full exploitation of these systems in ways that take full advantage of the tunable wetting and that can extend the patterned titania deposition previously reported [16] will be the subject of future investigations.

## Supporting Information

File 1 Isolated yields, ^1^H and ^13^C NMR, and exact mass MS data for the olefin-thioester precursors of compounds **1–4**.
