# Supplementary material for: Variations in the structure and reactivity of thioester functionalized self-assembled monolayers and their use for controlled surface modification
Source: Beilstein J Nanotechnol. 2012 Mar 9;3:213–20. doi: 10.3762/bjnano.3.24 (PMC3323910; doi:10.3762/bjnano.3.24)
Supplement: File 1 — Isolated yields, 1H and 13C NMR, and exact mass MS data for the olefin-thioester precursors of compounds 1–4. [file Beilstein_J_Nanotechnol-03-213-s001.pdf]

Supporting Information

for

# Variations in the structure and reactivity of thioester functionalized self-assembled monolayers and their use for controlled surface modification

Inbal Aped, Yacov Mazuz, and Chaim N. Sukenik*

Address: Department of Chemistry and Institute for Nanotechnology and Advanced Materials, Bar-Ilan University, Ramat-Gan, Israel 52900

E-mail: Chaim Sukenik - chaim.sukenik@biu.ac.il
*corresponding author

## Isolated yields, $^{1}H$ and $^{13}C$ NMR, and exact mass MS data for the olefin-thioester precursors of compounds 1–4.

**ω-Undecenyl thiopropionate**: yield 1.9 g (73%).

**$^1$H NMR**: 1.15 (t, *J* = 7.5 Hz, 3H), 1.26–1.37 (m, 12H), 1.54 (m, 2H), 2.02 (m, 2H), 2.54 (q, *J* = 7.5 Hz, 2H), 2.84 (t, *J* = 7.5, 2H), 4.88–5.05 (m, 2H), 5.81 (ddt, *J* = 6.6, 10.2, 17 Hz, 1H);

**$^{13}$C NMR**: 9.81, 28.85, 28.93, 29.01, 29.19 (2C), 29.50 (2C), 29.70, 33.9, 37.52, 114.22, 139.25, 200.47.

**MS**: $MH^+$ $C_{14}H_{27}OS$; 243.1777 (found), 243.1783 (calculated);

**ω-Undecenyl thiobutyrate**: yield (76%).

**$^1$H NMR**: 0.94 (t, *J* = 7.5 Hz, 3H), 1.26–1.37 (m, 12H), 1.54 (m, 2H), 1.67 (sextet, *J* = 7.5 Hz, 2H), 2.03 (m, 2H), 2.5 (t, *J* = 7.5 Hz, 2H), 2.84 (t, *J* = 7.5 Hz, 2H), 4.88–5.05 (m, 2H), 5.81 (ddt, *J* = 6.6, 10.2, 17 Hz, 1H);

**$^{13}$C NMR**: 13.6, 19.33, 28.89, 28.92, 29.02, 29.2 (2C), 29.50 (2C), 29.72, 33.91, 46.09, 114.22, 139.29, 199.74.

**MS**: $MH^+$ $C_{15}H_{29}OS$; 257.1894 (found), 257.1939 (calculated);

$M^+$ $C_{15}H_{28}OS$; 256.1836 (found), 256.1861 (calculated);

**ω-Undecenyl thiopentanoate**: yield (80%).

**$^1$H NMR**: 0.9 (t, *J* = 7.5 Hz, 3H), 1.26–1.37 (m, 14H), 1.54 (m, 2H), 1.62 (m, 2H), 2.02 (m, 2H), 2.52 (t, *J* = 7.5 Hz, 2H), 2.84 (t, *J* = 7.5, 2H), 4.88–5.05 (m, 2H), 5.81 (ddt, *J* = 6.6, 10.2, 17 Hz, 1H);

**$^{13}$C NMR**: 13.83, 22.23, 27.89, 28.93 (2C), 29.03, 29.21 (2C), 29.51 (2C), 29.72, 33.92, 43.98, 114.23, 139.29, 199.86.

**MS**: $MH^+$ $C_{16}H_{31}OS$; 271.2063 (found), 271.2096 (calculated);

$M^+$ $C_{16}H_{30}OS$; 270.1982 (found), 270.2017 (calculated);

**ω-Undecenyl thiohexanoate**: yield (76%).

**$^1$H NMR**: 0.87 (t, *J* = 7.5 Hz, 3H), 1.26–1.37 (m, 16H), 1.54 (m, 2H), 1.64 (m, 2H), 2.02 (m, 2H), 2.51 (t, *J* = 7.5 Hz, 2H), 2.84 (t, *J* = 7.5, 2H), 4.88–5.05 (m, 2H), 5.81 (ddt, *J* = 6.6, 10.2, 17 Hz, 1H);

$^{13}$**C NMR**: 13.97, 22.43, 25.5, 28.92 (2C), 29.02, 29.2 (2C), 29.51 (2C), 29.71, 31.22, 33.9, 44.21, 114.22, 139.26, 199.85.

**MS**: $MH^+$ $C_{17}H_{33}OS$; 285.2251 (found), 285.2252 (calculated);

**ω-Undecenyl thioheptanoate**: yield (85%).

$^1$**H NMR**: 0.87 (t, *J* = 7.5 Hz, 3H), 1.26–1.37 (m, 18H), 1.54 (m, 2H), 1.64 (m, 2H), 2.02 (m, 2H), 2.51 (t, *J* = 7.5 Hz, 2H), 2.84 (t, *J* = 7.5, 2H), 4.88–5.05 (m, 2H), 5.81 (ddt, *J* = 6.6, 10.2, 17 Hz, 1H);

$^{13}$**C NMR**: 13.93, 22.43, 25.6, 28.56, 28.72, 28.75, 28.86, 29.03 (2C), 29.33, 29.36, 29.55, 31.39, 33.74, 44.08, 114.05, 139.07, 199.61.

**MS**: $MH^+$ $C_{18}H_{35}OS$; 299.2382 (found), 299.2409 (calculated);

**ω-Undecenyl thiooctanoate**: yield (71%).

$^1$**H NMR**: 0.87 (t, *J* = 7.5 Hz, 3H), 1.26–1.37 (m, 20H), 1.54 (m, 2H), 1.64 (m, 2H), 2.02 (m, 2H), 2.51 (t, *J* = 7.5 Hz, 2H), 2.84 (t, *J* = 7.5, 2H), 4.88–5.05 (m, 2H), 5.81 (ddt, *J* = 6.6, 10.2, 17 Hz, 1H);

$^{13}$**C NMR**: 14.17, 22.71, 25.84, 28.93 (2C), 29.04 (3C), 29.22 (2C), 29.53 (2C), 29.72, 31.75, 33.92, 44.27, 114.23, 139.28, 199.87.

**MS**: $MH^+$ $C_{19}H_{37}OS$; 313.2569 (found), 313.2565 (calculated);

**ω-Undecenyl thiononanoate**: yield (81%).

$^1$**H NMR**: 0.86 (t, *J* = 7.5 Hz, 3H), 1.26–1.37 (m, 22H), 1.54 (m, 2H), 1.64 (m, 2H), 2.02 (m, 2H), 2.51 (t, *J* = 7.5 Hz, 2H), 2.84 (t, *J* = 7.5, 2H), 4.88–5.05 (m, 2H), 5.81 (ddt, *J* = 6.6, 10.2, 17 Hz, 1H);

$^{13}$**C NMR**: 14.19, 22.75, 25.83, 28.91, 28.93, 29.03, 29.07, 29.2 (3C), 29.33 29.53 (2C), 29.72, 31.91, 33.92, 44.27, 114.23, 139.27, 199.87.

**MS**: $MH^+$ $C_{20}H_{39}OS$; 327.2766 (found), 327.2722 (calculated);

**ω-Undecenyl thiodecanoate**: yield (74%).

**$^{1}$H NMR**: 0.86 (t, *J* = 7.5 Hz, 3H), 1.26–1.37 (m, 24H), 1.54 (m, 2H), 1.64 (m, 2H), 2.02 (m, 2H), 2.51 (t, *J* = 7.5 Hz, 2H), 2.84 (t, *J* = 7.5, 2H), 4.88–5.05 (m, 2H), 5.81 (ddt, *J* = 6.6, 10.2, 17 Hz, 1H);

**$^{13}$C NMR**: 14.24, 22.8, 25.87, 28.95 (2C), 29.06, 29.1, 29.24 (2C), 29.39 (2C) 29.53 (3C), 29.73, 31.99, 33.94, 44.31, 114.25, 139.35, 200.01.

**MS**: $MH^+$ $C_{21}H_{41}OS$; 341.2892 (found), 341.2878 (calculated);

$M^+$ $C_{16}H_{30}OS$; 270.1982 (found), 270.2017 (calculated);

***S*-undec-10-enyl benzothioate**: yield (76%). NMR taken in Bruker DPX 200 spectrometer.

**$^{1}$H NMR**: 1.13–1.51 (m, 12H), 1.67 (m, 2H), 2.03 (m, 2H), 3.06 (t, *J* = 7.2 Hz, 2H), 4.88–5.05 (m, 2H), 5.81 (ddt, *J* = 6.6, 10.2, 17 Hz, 1H), 7.42 (m, 2H), 7.55 (tt, *J* = 1.4, 7.3 Hz, 1H), 7.97 (m, 2H);

**$^{13}$C NMR**: 29.04 (2C), 29.12, 29.20, 29.23, 29.52 (2C), 29.66, 33.93, 114.23, 127.28 (2C), 128.63 (2C), 133.27, 137.39, 139.27, 192.16;

**MS**: $MH^+$ $C_{18}H_{27}OS$; 291.1760 (found), 291.4760 (calculated).

***S*-undec-10-enyl thiophene-3-carbothioate**: yield (70%). NMR taken in Bruker DPX 200 spectrometer. Mass spectrum was recorded on a Q-Tof micro (Waters U.K.) using ESI.

**$^{1}$H NMR**: 1.18–1.50 (m, 12H), 1.66 (m, 2H), 2.04 (m, 2H), 3.04 (t, *J* = 7.1 Hz, 2H), 4.88–5.05 (m, 2H), 5.81 (ddt, *J* = 6.6, 10.2, 17 Hz, 1H), 7.31 (dd, *J* = 3, 5.1 Hz, 1H), 7.53 (dd, *J* = 1.3, 5.1 Hz, 1H), 8.09 (dd, *J* = 1.3, 3 Hz, 1H);

**$^{13}$C NMR**: 28.99 (3C), 29.22 (2C), 29.52 (2C), 29.76, 33.92, 114.25, 126.13, 126.40, 130.32, 139.31, 141.29, 185.81;

**MS**: $MH^+$ $C_{16}H_{25}OS_2$; 297 (found), 297.5050 (calculated).

***S*-undec-10-enyl thiophene-2-carbothioate**: yield (80%). NMR taken in Bruker DPX 200 spectrometer. Mass spectrum was recorded on a Q-Tof micro (Waters U.K.) using ESI.

**$^{1}$H NMR**: 1.21–1.50 (m, 12H), 1.67 (m, 2H), 2.04 (m, 2H), 3.06 (t, *J* = 7.1 Hz, 2H), 4.88–5.05 (m, 2H), 5.81 (ddt, *J* = 6.6, 10.2, 17 Hz, 1H), 7.10 (dd, *J* = 3.85, 5. Hz, 1H), 7.59 (dd, *J* = 1.15, 5 Hz, 1H), 7.79 (dd, *J* = 1.15, 3.85 Hz, 1H);
**$^{13}$C NMR**: 28.97, 29.04, 29.22 (2C), 29.33, 29.53 (2C), 29.77, 33.93, 114.25, 127.93, 130.94, 132.47, 139.34, 142.52, 184.28;
**MS**: $MH^+$ $C_{16}H_{25}OS_2$; 297 (found), 297.5050 (calculated).

The isolated yields and NMR data for each of the trichlorosilanes is as follows:

**1a**: 60 °C and 16 h, yield (90%).
**$^{1}$H NMR**: 1.16–1.47 (m, 16H), 1.56 (m, 4H), 2.32 (s, 3H), 2.86 (t, *J* = 7.1 Hz, 2H);
**$^{13}$C NMR**: 22.37, 24.43, 28.93, 29.11, 29.21, 29.28, 29.42, 29.53, 29.61 (2C), 30.76, 31.93, 196.2401.

**1b**: 60 °C and 16 h, yield (80%).
**$^{1}$H NMR**: 1.17 (t, *J* = 7.5 Hz, 3H), 1.26–1.37 (m, 16H), 1.54 (m, 4H), 2.55 (q, *J* = 7.5 Hz, 2H), 2.84 (t, *J* = 7.5 Hz, 2H);
**$^{13}$C NMR**: 9.72, 22.22, 24.28, 28.73, 28.8, 28.96, 29.06, 29.27, 29.38, 29.44, 29.57, 31.77, 37.42, 200.52.

**1c**: 60 °C and 16 h, yield (82%).

**$^{1}$H NMR**: 0.94 (t, *J* = 7.5 Hz, 3H), 1.26–1.37 (m, 16H), 1.54 (m, 4H), 1.68 (sexth, *J* = 7.5 Hz, 2H), 2.51 (t, *J* = 7.5 Hz, 2H), 2.85 (t, *J* = 7.5 Hz, 2H);
**$^{13}$C NMR**: 13.47, 19.2, 22.22, 24.27, 28.75, 28.78, 28.95, 29.06, 29.27, 29.38, 29.44, 29.57, 31.77, 45.95, 199.71.

**1d**: 60 °C and 16 h, yield (78%).

**$^{1}$H NMR**: 0.9 (t, *J* = 7.5 Hz, 3H), 1.26–1.37 (m, 18H), 1.5–1.68 (m, 6H), 2.53 (t, *J* = 7.5 Hz, 2H), 2.85 (t, *J* = 7.5 Hz, 2H);

**$^{13}$C NMR**: 13.71, 22.09, 22.22, 24.28, 27.76, 28.78 (2C), 28.96, 29.06, 29.28, 29.38, 29.45, 29.57, 31.78, 43.85, 199.89.

**1e**: 60 °C and 16 h, yield (68%).

**$^{1}$H NMR**: 0.89 (m, 3H), 1.26–1.37 (m, 20H), 1.5–1.68 (m, 6H), 2.53 (t, $J$ = 7.5 Hz, 2H), 2.85 (t, $J$ = 7.5 Hz, 2H);

**$^{13}$C NMR**: 13.86, 22.22, 22.31, 24.28, 25.4, 28.8 (2C), 28.97, 29.07, 29.28, 29.39, 29.45, 29.56, 31.09, 31.78, 44.1, 200.08.

**1f**: 60 °C and 16 h, yield (82%).

**$^{1}$H NMR**: 0.88 (m, 3H), 1.26–1.37 (m, 22H), 1.5–1.68 (m, 6H), 2.53 (t, $J$ = 7.5 Hz, 2H), 2.85 (t, $J$ = 7.5 Hz, 2H);

**$^{13}$C NMR**: 14.01, 22.23, 22.44, 24.29, 25.68, 28.61, 28.8 (2C), 28.97, 29.08, 29.29, 29.4, 29.46, 29.58, 31.43, 31.79, 44.15, 199.96.

**1g**: 60 °C and 16 h, yield (69%).

**$^{1}$H NMR**: 0.88 (m, 3H), 1.26–1.37 (m, 24H), 1.5–1.68 (m, 6H), 2.53 (t, $J$ = 7.5 Hz, 2H), 2.85 (t, $J$ = 7.5 Hz, 2H);

**$^{13}$C NMR**: 14.05, 22.23, 22.57, 24.28, 25.72, 28.79 (2C), 28.9 (2C), 28.97, 29.07, 29.28, 29.39, 29.45, 29.56, 31.61, 31.78, 44.14, 200.04.

**1h**: 60 °C and 16 h, yield (86%).

**$^{1}$H NMR**: 0.87 (m, 3H), 1.26–1.37 (m, 26H), 1.5–1.68 (m, 6H), 2.52 (t, $J$ = 7.5 Hz, 2H), 2.85 (t, $J$ = 7.5 Hz, 2H);

**$^{13}$C NMR**: 14.07, 22.22, 22.61, 24.27, 25.69, 28.78 (2C), 28.93, 28.96, 29.06 (2C), 29.19, 29.28, 29.39, 29.45, 29.57, 31.77 (2C), 44.12, 199.84.

**1i**: 60 °C and 16 h, yield (79%).

**$^{1}$H NMR**: 0.87 (m, 3H), 1.26–1.37 (m, 28H), 1.5–1.68 (m, 6H), 2.52 (t, *J* = 7.5 Hz, 2H), 2.85 (t, *J* = 7.5 Hz, 2H);
**$^{13}$C NMR**: 14.09, 22.22, 22.62, 24.28, 25.71, 28.79 (2C), 28.93, 28.96, 29.07, 29.24 (2C), 29.28, 29.36, 29.39, 29.45, 29.58, 31.79, 31.83, 44.13, 199.88.

**2**: 60 °C and 21 h, yield (90%).

**$^{1}$H NMR**: 1.22–1.49 (m, 16H), 1.52–1.73 (m, 4H), 3.07 (t, *J* = 7.4 Hz, 2H), 7.44 (m, 2H), 7.56 (tt, *J* = 1.4, 7.3 Hz, 1H), 7.97 (m, 2H);
**$^{13}$C NMR**: 22.39, 24.45, 29.05, 29.13, 29.19, 29.26, 29.45, 29.56, 29.62, 29.70, 31.94, 127.32 (2C), 128.69 (2C), 133.34, 137.42, 192.29.

**3**: 80 °C and 40 h, yield (90%).

**$^{1}$H NMR**: 1.22–1.47 (m, 16H), 1.52–1.72 (m, 4H), 3.04 (t, *J* = 7.3 Hz, 2H), 7.32 (dd, *J* = 3, 5.1 Hz, 1H), 7.53 (dd, *J* = 1.3, 5.1 Hz, 1H), 8.10 (dd, *J* = 1.3, 3 Hz, 1H);
**$^{13}$C NMR**: 22.25, 24.31, 28.87 (2C), 28.98, 29.11, 29.30, 29.41, 29.47, 29.64, 31.80, 126.02, 126.30, 130.21, 141.18, 185.75.

**4**: 80 °C and 40 h, yield (90%).

**$^{1}$H NMR**: 1.26–1.47 (m, 16H), 1.51–1.73 (m, 4H), 3.06 (t, *J* = 7.3 Hz, 2H), 7.10 (dd, *J* = 3.85, 5. Hz, 1H), 7.59 (dd, *J* = 1.15, 5 Hz, 1H), 7.79 (dd, *J* = 1.15, 3.85 Hz, 1H);
**$^{13}$C NMR**: 22.39, 24.44, 28.96, 29.12, 29.23, 29.34, 29.43, 29.54, 29.60, 29.79, 31.93, 127.95, 130.95, 132.49, 142.52, 184.29.
